# Rapid Identification of Relevant Microbial Strains by Identifying Multiple Marker Single Nucleotide Polymorphisms via Amplicon Sequencing: Epidemic Monkeypox Virus as a Proof of Concept

**DOI:** 10.1128/spectrum.04196-22

**Published:** 2023-01-05

**Authors:** Sergio Buenestado-Serrano, Marta Herranz, Rosalía Palomino-Cabrera, Cristina Rodríguez-Grande, Daniel Peñas-Utrilla, Andrea Molero-Salinas, Cristina Veintimilla, Pilar Catalán, Roberto Alonso, Patricia Muñoz, Laura Pérez-Lago, Darío García de Viedma

**Affiliations:** a Servicio de Microbiología Clínica y Enfermedades Infecciosas, Gregorio Marañón General University Hospital, Madrid, Spain; b Instituto de Investigación Sanitaria Gregorio Marañón, Madrid, Spain; c Escuela de Doctorado, Universidad de Alcalá, Alcalá de Henares, Madrid, Spain; d CIBER de Enfermedades Respiratorias, Instituto de Salud Carlos III (CIBERES), Madrid, Spain; e Departamento de Medicina, Universidad Complutense, Madrid, Spain; Institute of Molecular Biology, Academia Sinica

**Keywords:** MPXV, monkeypox virus, nanopore, amplicons, sequencing

## Abstract

Despite the proven value of applying genomic data for epidemiological purposes, commonly used high-throughput sequencing formats are not adapted to the response times required to intervene and finally control outbreaks. In this study, we propose a fast alternative to whole-genome sequencing (WGS) to track relevant microbiological strains: nanopore sequencing of multiple amplicons including strain marker single nucleotide polymorphisms (SNPs). As a proof a concept, we evaluated the performance of our approach to offer a rapid response to the most recent public health global alarm, the monkeypox virus (MPXV) global outbreak. Through a multisequence alignment, a list of 42 SNPs were extracted as signature makers for this outbreak. Twenty primer pairs were designed to amplify in a multiplex PCR the regions including 22 of these SNPs. Amplicon pools were sequenced in a MinION device, and SNPs were called in real time by an in-house bioinformatic pipeline. A total of 120 specimens (95 MPXV-PCR positive, Ct values from 14 to 39) were selected. In 67.37% of the positive subset, all 22 SNPs were called. After excluding low viral load specimens, in 92% of samples ≥11 outbreak SNPs were called. No false positives were observed in any of the 25 negative specimens. The total turnaround time required for this strategy was 5 hours, and the cost per sample was 14 euros. Nanopore sequencing of multiple amplicons harboring signature SNPs escapes the targeting limitations of strain-specific PCRs and offers a powerful alternative to systematic WGS, paving the way to real-time genomic epidemiology and making immediate intervention possible to finally optimize transmission control.

**IMPORTANCE** Nanopore sequencing of multiple amplicons harboring signature single nucleotide polymorphisms (SNPs) escapes the targeting limitations of strain-specific PCRs and offers a powerful alternative to systematic whole-genome analysis, paving the way to real-time genomic epidemiology and making immediate intervention possible to finally optimize transmission control.

## OBSERVATION

The COVID-19 pandemic has demonstrated how valuable systematically obtained genomic data are to track the global dispersion of emerging SARS-CoV-2 variants (1). Similarly, rapid genomic analysis after the first cases of the monkeypox virus (MPXV) outbreak were diagnosed in Europe allowed us to determine the most likely source in Africa from whence the cases were initially exported before spreading across Europe. The exploitation of subsequent data identified the genomic signatures shared by the cases involved in this outbreak variant, including 42 mutations not present in its most recent ancestor. Genomic epidemiological analysis has proven to be equally valuable to track the transmission of many other pathogens, both at a population context level and in a nosocomial setting (2–4).

Despite the proven value of applying genomic data to epidemiological purposes, we must acknowledge that the analysis times associated with commonly used high-throughput formats are not adapted to the real-time identification of epidemiologically relevant strains that would be required to pursue maximum efficiency to intervene and finally control outbreaks.

To accelerate response times and reduce costs, we previously proposed an alternative to systematic genomic analysis to track the transmission of Mycobacterium tuberculosis high-risk strains (5). Our strategy was based on (i) identifying the more active transmission clusters based on molecular analysis (VNTR [Variable Number of Tandem Repeats]), (ii) WGS analysis of several representatives of the clusters to be preferentially surveyed, (iii) identifying specific single nucleotide polymorphism (SNP) markers for the strains involved, and (iv) designing multiplex allele-specific PCRs to target strain-specific markers. Based on the prospective application of these PCRs, we succeeded in optimizing the surveillance of tuberculosis transmission hot spots in Spain, France, Panama, Argentina, Costa Rica, and Morocco (6–8).

In this study, we aimed to maintain the same philosophy while improving its performance by markedly increasing the number of specific SNPs simultaneously targeted, which constituted the bottleneck of our previous multiplex-PCR-based approach. To address this, we transferred our initial strategy to multiple-amplicon rapid sequencing in nanopores using the MinION system (Oxford Nanopore Technologies, Oxford, UK). As a proof of concept, we evaluated the performance of our approach in offering a rapid response to the most recent current public health global alarm, the MPXV global outbreak, during which Spain has accumulated the third highest number of cases in the world, according to the Centers for Disease Control and Prevention.

First, we downloaded the 54 current outbreak genomes available at the NCBI and Virological.org up to 2 June 2022, as well as two references from the 2019 outbreak and two representative sequences from clades I and II. Using MAFFT (9) with an FFT-NS-2 strategy, we performed a multiple sequence alignment of these genomes, keeping the SNPs shared among the 2022 outbreak sequences but absent in their most recent ancestor strains, preceding the outbreak. The final list of 42 SNPs considered by our analysis to be the signature markers for this outbreak also corresponded to those proposed elsewhere (2).

Second, we designed 20 primer sets to obtain 320- to 340-bp amplicons, which included 22 of the 33 signature SNPs (9 SNPs were discarded for being part of tandem repeat sequences located at the ends of the viral genome); each amplicon included one marker SNP, except two that included two markers (Table 1). Primer sequences were designed to share the same PCR conditions so as to multiplex them in the same reaction. The PCR conditions were as follows: 2.5 mM MgCl_2_, 0.2 μM each primer, 200 μM dNTPs, and 0.4 μL AmpliTaq Gold (Applied Biosystems, Foster City, CA, USA) in a final reaction volume of 25 μL. The PCR was run at 95°C for 5 min, followed by 30 cycles (95°C for 1 min, 60°C for 1 min, and 72°C for 1 min), and a final extension step at 72°C for 10 min. A pool of 20 amplicons from each positive MPXV specimen, as per the LightMix modular monkeypox virus kit (Roche, Basel, Switzerland), was used to prepare the sequencing library using a rapid barcoding kit (SQK-RBK110-96, Oxford Nanopore Technologies, Oxford, UK), which was loaded in a Minion device (Flow cell r9.4.1). A pipeline (https://github.com/MG-IiSGM/virION) was prepared for real-time analysis of the sequences obtained from the nanopore sequencing. The pipeline first preprocessed the fast5 raw files into FastQ files; then aligned them against the MPXV-UK_P2 (MT903344.1), an ancestral strain for the outbreak; and finally called the 22 SNPs selected as specific for the outbreak strain.

**TABLE 1:** Primer sequences for the multiplex PCR and SNP associated with each amplicon

SNP^*a*^ ID	SNP position^*b*^	Primer ID	Primer sequence
SNP6	C7780T	SNP6_MPXV_F	TCTGATGTTGTTGTTCGCTGC
		SNP6_MPXV_R	TGCCTACTAATACAAGCACAATACC
SNP10	G30376A	SNP10_MPXV_F	CGGCATATTAACCCAAGCAGC
		SNP10_MPXV_R	ACAACCACGCAAATCATACAAGT
SNP11	G31062A	SNP11_MPXV_F	AGAAGTTAGACTGTGAATGTCAAGG
		SNP11_MPXV_R	CGAAAAACGTGTGGGTGAATACC
SNP12	G34468A	SNP12_MPXV_F	AACCACAATTTGCTTCCGCC
		SNP12_MPXV_R	TAAAAATTGCGCGCTCCGAA
SNP13	G38369A	SNP13_MPXV_F	CATCCACAGTTATTGGGCCAG
		SNP13_MPXV_R	GCGTCCCGAGATTGATGTGT
SNP15	C39148T	SNP15_MPXV_F	GCTGTTTTCATCAAGGTTTGTATCC
		SNP15_MPXV_R	CGCCAGAAGTCTAGATGCGT
SNP17	G54126A	SNP17_MPXV_F	TGTCAAATATTGCTCGTCCAACG
		SNP17_MPXV_R	GTCGAAATGGCGAGACCGTA
SNP18	G54644A	SNP18_MPXV_F	AAACATTGGGGAAGAGCCGT
		SNP18_MPXV_R	ACGCGGTTATTTCAGTCGTG
SNP19	G64306A	SNP19_MPXV_F	TCCACTTCGGATCCATAAGCAA
		SNP19_MPXV_R	GCACCTGAGCAGACCCAAT
SNPs20/21	C73075T/G73248A	SNP20_21_MPXV_F	AATCGATAAATTGCGCCAAATTGTG
		SNP20_21_MPXV_R	TGTCCTAATAGGCATCAGTTCCTTG
SNP22	G74214A	SNP22_MPXV_F	ACCCAATTGCTGTCGCAC
		SNP22_MPXV_R	TGTGCGCGTAAATGATGCAA
SNP23	G77392A	SNP23_MPXV_F	CTTCCGTCAAGGATCATCACCTA
		SNP23_MPXV_R	AACACATTCCAATAGTCCCAGAAAA
SNP25/26	C82382T/G82460A	SNP25_26_MPXV_F	TTGCCTGGTGATTGGGTAGAA
		SNP25_26_MPXV_R	AAACAATGAATACGCTGCTACGA
SNP27	G95043A	SNP27_MPXV_F	CTGGAAGACAAGTTAGATGGTGT
		SNP27_MPXV_R	TTCTTCGGTATCCTTGTATCTAACT
SNP28	G124139A	SNP28_MPXV_F	ATGAAGACGGCGATTTCGTAGA
		SNP28_MPXV_R	TTTTCTGCCGTGTGTGGTCA
SNP29	G124683A	SNP29_MPXV_F	TCCGGATGTATTAATCGTAGTCAGT
		SNP29_MPXV_R	GGTCCATCTAGTCGTTTTACCA
SNP30	C128707T	SNP30_MPXV_F	ATGTACTCATGGCTACGGCATT
		SNP30_MPXV_R	GACGATCTACCGATTCCGTTT
SNP32	A151472C	SNP32_MPXV_F	ATGATGTTACCCGATCCTCTCTT
		SNP32_MPXV_R	CTGTGGATCGCTGAGGAAAGT
SNP36	G181995A	SNP36_MPXV_F	CAGCAGTTCCTTATGATCAACGAT
		SNP36_MPXV_R	TGGTGGAAGATATGAAGGTGTCG
SNP37	C183534T	SNP37_MPXV_F	ACGCCCTACAAATGATGGTCT
		SNP37_MPXV_R	ACCAACCTCCACATATTCTGGT

a SNP, single nucleotide polymorphism.

b Based on MT903344.1 reference.

For our evaluation, we selected PCR-positive and -negative specimens from cases with an MPXV infection suspicion received in our institution for reverse transcription (RT)-PCR testing. Between June 21 and September 16, 2022, a total of 120 specimens (104 cutaneous lesions, 5 rectal exudates, 3 rectal/pharyngeal pools, 3 plasma, 2 pharyngeal exudates, 2 abscesses, and 1 cerebrospinal fluid [CSF]) were selected. Among these, 95 samples from 87 different patients were RT-PCR-positive (Ct values ranging from 14 to 39). The specimens were sequenced and run in 12 independent MinION runs (10 specimens per run were loaded, using a different set of indexes per run).

In 64 specimens (67.37% of the positive subset), all 22 SNPs were called at ≥*30X depth* (average coverage (477.72 times) after 60 min of running (to ensure a response time similar to that required for an RT-PCR assay). If we lowered the threshold for the number of SNPs properly called to 12 (≥*20X depth*), we increased the number of MPXV identifications to 73 (77%). Among the remaining specimens, after having eliminated 10 with low viral loads (Ct value >30), with 5 called ≥11 SNPs (≥*12X depth*). In summary, after excluding low viral load specimens, 92% of samples assayed called a sufficiently high set of specific MPXV outbreak SNPs. In all but 2 of the 25 PCR-negative specimens, no SNPs were called; a single SNP, with very low coverage (>*5X depth*), was called in two specimens.

It could be considered a limitation that not all targeted SNPs were called in some of the specimens with low viral loads. We must consider that, first, 80% of the total 644 specimens with a positive MPXV PCR in our laboratory presented Ct values <29; therefore, specimens with lower viral loads were rather infrequent, most corresponding to plasma samples. Second, we evaluated whether the reduction in the number of SNPs called in low viral load specimens could lead to misidentifications of the MXPV outbreak strain. To this end, we performed an *in-silico* evaluation of the number of targeted SNPs that would be called in other monkeypox viruses. We downloaded 20 FastQ files from monkeypox virus (4 from 2019 and 16 from 2020). None of the targeted SNPs were called, except for one SNP in one case. When proceeding similarly with FastQ files from other orthopoxviruses (camelpox, cowpox, vaccinia, variola, etc.), marked differences were found in the sequences flanked by the 20 primer sets used in our approach, and therefore, potential misidentifications were fully ruled out. All these data together signify that, even when supporting our identification on the calling of a subset of the total SNPs targeted, the identification of the MPXV outbreak strain is highly specific.

In five specimens, we identified one or two private SNPs in the regions analyzed in our strategy. This indicated that with just 3.3% of the genome sequenced, we could capture some of the diversity acquired by MPXV during this outbreak (Public Health England (PHE) technical briefing 7).

The systematic application of RT-PCR in COVID-19 diagnosis has taught us the usefulness of having an indirect inference of viral load, based on Ct values (10). Similarly, we aimed to evaluate whether we could extract some inference regarding MPXV viral load in the specimen from our nanopore sequencing data. For each specimen, we extracted several parameters from the sequencing run: final average coverage, number of SNPs called at ≥*30X depth*, and number of active pores/sequencing pores in the flow cell. From these values, we defined a model of multiple linear regression. When we compared the experimental Ct values obtained by RT-PCR for each specimen with the values inferred from our model, we observed a deviation of ±3 (Table 2). This inference was far from being precise, but it could still be useful to categorize each specimen into a range of low/intermediate/high viral load.

**TABLE 2:** Results obtained in the project^*a*^

Sample	ENA	Sample	SNPs			Mean stats		Ct		
			Called	Outbreak	>30X depth	Coverage	Frequency	PCR	Inferred	Difference
6617233	ERS13545281	Cutaneous lesion	22	22	22	974	0.96	20	20.00	0.00
6617225	ERS13545282	Cutaneous lesion	22	22	16	43.35	0.97	24	25.38	1.38
6617224	ERS13545283	Cutaneous lesion	22	22	22	114.35	0.97	23	23.74	0.74
6617273	ERS13545284	Cutaneous lesion	22	22	21	81.7	0.96	25	24.10	−0.90
6617344	ERS13545285	Cutaneous lesion	22	22	4	19.48	0.98	26	28.15	2.15
6624173	ERS13545286	Cutaneous lesion	22	22	22	1,142.04	0.96	16	19.27	3.27
6627502	ERS13545287	Cutaneous lesion	22	22	22	727.78	0.96	21	21.07	0.07
6617279	ERS13545288	Cutaneous lesion	22	22	22	1,392.57	0.96	19	18.18	−0.82
6624186	ERS13545289	Cutaneous lesion	0	0	0	0	0	NEG		
6617222	ERS13545290	Cutaneous lesion	0	0	0	0	0	NEG		
6628559	ERS13545291	Cutaneous lesion	22	22	22	527.35	0.97	22	21.62	−0.38
6610604	ERS13545292	Cutaneous lesion	0	0	0	0	0	NEG		
6628537	ERS13545293	Abscesses	0	0	0	0	0	NEG		
6617380	ERS13545294	Cutaneous lesion	22	22	22	1,381.22	0.96	16	17.90	1.90
6617361	ERS13545295	Cutaneous lesion	22	22	22	797.26	0.96	19	20.44	1.44
6617362	ERS13545296	Cutaneous lesion	22	22	22	713.39	0.97	20	20.81	0.81
6617363	ERS13545297	Cutaneous lesion	22	22	20	60.83	0.97	24	24.09	0.09
6628926	ERS13545298	Cutaneous lesion	22	22	22	1,157.09	0.96	17	18.88	1.88
6628925	ERS13545299	Rectal/pharyngeal	14	14	0	9.07	1	29	28.76	−0.24
6628927	ERS13545300	Rectal/pharyngeal	0	0	0	0	0	36		
6628164	ERS13545301	Cutaneous lesion	1	1	0	6	0.83	39		
6617307	ERS13545302	Cutaneous lesion	1	1	0	5	1	NEG		
6617269	ERS13545303	Plasma	0	0	0	0	0	32		
6617395	ERS13545304	Cutaneous lesion	0	0	0	0	0	NEG		
6617314	ERS13545305	Cutaneous lesion	0	0	0	0	0	NEG		
6628217	ERS13545306	Cutaneous lesion	22	22	22	1,019.39	0.96	19	19.15	0.15
6628164-5	ERS13545307	Cutaneous lesion	0	0	0	0	0	39		
6617307-5	ERS13545308	Cutaneous lesion	1	1	0	5	1	NEG		
6617269-5	ERS13545309	Plasma	3	3	0	7.67	0.91	32		
6628217-5	ERS13545310	Cutaneous lesion	21	21	20	1427	0.96	19	17.83	−1.17
6621312	ERS13545311	Rectal Exudates	22	22	22	655.09	0.97	29,73	20.42	−9.31
6622760	ERS13545312	Rectal exudates	22	22	22	361.36	0.96	18,4	21.69	3.29
6628226	ERS13545313	Cutaneous lesion	6	6	0	6.83	1	27	28.13	1.13
6628227	ERS13545314	Cutaneous lesion	16	16	1	15.25	0.98	31		
6628228	ERS13545315	Cutaneous lesion	22	22	22	559.64	0.96	19	20.83	1.83
6628235	ERS13545316	Cutaneous lesion	22	22	22	469.77	0.96	18	21.22	3.22
6617282	ERS13545317	Cutaneous lesion	22	22	22	164.95	0.96	24	22.55	−1.45
6617283	ERS13545318	Cutaneous lesion	22	22	22	443.86	0.97	19	21.33	2.33
6628236	ERS13545319	Cutaneous lesion	0	0	0	0	0	NEG		
6628237	ERS13545320	Cutaneous lesion	0	0	0	0	0	NEG		
6628235-5	ERS13545321	Cutaneous lesion	22	22	22	1,002.05	0.96	18	18.58	0.58
6628236-5	ERS13545322	Cutaneous lesion	0	0	0	0	0	NEG		
6628237-5	ERS13545323	Cutaneous lesion	0	0	0	0	0	NEG		
6617207	ERS13545324	Cutaneous lesion	22	22	22	1,071.09	0.96	19	18.28	−0.72
6617270	ERS13545325	Cutaneous lesion	22	22	22	697.59	0.96	22	19.91	−2.09
6617271	ERS13545326	Cutaneous lesion	22	22	22	1,320.27	0.96	23	17.20	−5.80
6617113	ERS13545327	Cutaneous lesion	22	22	22	738.55	0.96	21	19.73	−1.27
6613821	ERS13545328	Cutaneous lesion	20	20	3	19.5	0.97	26	27.08	1.08
6613834	ERS13545329	Cutaneous lesion	22	22	22	542.68	0.96	25	20.58	−4.42
6613837	ERS13545330	Cutaneous lesion	22	22	22	625.27	0.97	20	20.22	0.22
6628316	ERS13545331	Cutaneous lesion	22	22	6	23.27	0.96	28	26.08	−1.92
6637422	ERS13545332	Cerebrospinal fluid	0	0	0	0	0	29	27.51	−1.49
6613834-5	ERS13545333	Cutaneous lesion	22	22	22	151.14	0.96	25	21.96	−3.04
6613837-5	ERS13545334	Cutaneous lesion	22	22	22	312.64	0.96	20	21.26	1.26
6610169	ERS13545335	Cutaneous lesion	22	22	22	295.5	0.96	21	21.34	0.34
6610160	ERS13545336	Cutaneous lesion	22	22	22	242.05	0.96	22	21.57	−0.43
6617250	ERS13545337	Cutaneous lesion	22	22	16	36.68	0.96	24.31	23.80	−0.51
6617251	ERS13545338	Cutaneous lesion	22	22	22	218.82	0.96	20.65	21.67	1.02
6610676	ERS13545339	Abscesses	0	0	0	0	0	NEG		
6617265	ERS13545340	Cutaneous lesion	0	0	0	0	0	NEG		
6613672	ERS13545341	Cutaneous lesion	22	22	22	238.59	0.96	25	21.26	−3.74
6613821-5	ERS13545342	Cutaneous lesion	12	12	0	7.92	0.97	26	27.16	1.16
6628333	ERS13545343	Cutaneous lesion	0	0	0	0	0	NEG		
6629444	ERS13545344	Cutaneous lesion	0	0	0	0	0	NEG		
6630612	ERS13545345	Cutaneous lesion	22	22	19	46.18	0.96	26	22.76	−3.24
6618985	ERS13545346	Cutaneous lesion	22	22	22	508.82	0.96	17	20.08	3.08
6617131	ERS13545347	Cutaneous lesion	20	20	0	13.8	0.97	28	27.13	−0.87
6608350	ERS13545348	Cutaneous lesion	22	22	22	163.77	0.96	22	21.59	−0.41
6628073	ERS13545349	Cutaneous lesion	22	22	20	49.45	0.95	24	22.53	−1.47
6618540	ERS13545350	Cutaneous lesion	23	22	22	943.3	0.96	16	18.19	2.19
6633875	ERS13545351	Cutaneous lesion	22	22	22	401.73	0.96	21	20.23	−0.77
6630369	ERS13545352	Cutaneous lesion	22	22	22	648.77	0.96	19	19.15	0.15
6630704	ERS13545353	Cutaneous lesion	0	0	0	0	0	NEG		
6630737	ERS13545354	Cutaneous lesion	0	0	0	0	0	NEG		
6633875-5	ERS13545355	Cutaneous lesion	22	22	6	24.59	0.96	24	25.43	1.43
6630743	ERS13545356	Cutaneous lesion	22	22	22	656.55	0.96	20	19.12	−0.88
6630976	ERS13545357	Cutaneous lesion	22	22	22	577.73	0.96	19	19.46	0.46
6630701	ERS13545358	Cutaneous lesion	8	8	0	9.88	0.96	27	26.82	−0.18
6630703	ERS13545359	Cutaneous lesion	0	0	0	0	0	32		
6636100	ERS13545360	Cutaneous lesion	22	22	22	259.09	0.96	23	20.85	−2.15
6747961	ERS13545361	Cutaneous lesion	22	22	22	345.05	0.96	20	20.15	0.15
6601275	ERS13545362	Cutaneous lesion	22	22	22	288.18	0.96	20	20.40	0.40
6601974	ERS13545363	Cutaneous lesion	22	22	22	280.45	0.95	30		
6747386	ERS13545364	Cutaneous lesion	22	22	22	543.77	0.96	15	19.29	4.29
6747387	ERS13545365	Rectal exudates	22	22	22	399.32	0.96	17	19.92	2.92
6740475	ERS13545366	Cutaneous lesion	0	0	0	0	0	NEG		
6630855	ERS13545367	Cutaneous lesion	0	0	0	0	0	38		
6699957	ERS13545368	Cutaneous lesion	22	22	22	150.32	0.96	22	21.00	−1.00
6699898	ERS13545369	Cutaneous lesion	22	22	22	80.41	0.96	23	21.30	−1.70
6601077	ERS13545370	Pharyngeal exudates	22	22	22	105.91	0.95	21	21.19	0.19
6630855-5	ERS13545371	Cutaneous lesion	0	0	0	0	0	NEG		
6630893	ERS13545372	Cutaneous lesion	0	0	0	0	0	NEG		
6747799	ERS13545373	Cutaneous lesion	24	22	22	124.58	0.96	18	20.79	2.79
6747765	ERS13545374	Cutaneous lesion	22	22	22	125.05	0.97	23	20.79	−2.21
6747766	ERS13545375	Cutaneous lesion	22	22	22	323.55	0.96	18	19.92	1.92
6740166	ERS13545376	Cutaneous lesion	3	3	0	6.33	0.86	27	26.19	−0.81
6747358	ERS13545377	Cutaneous lesion	22	22	22	247.55	0.96	18	20.25	2.25
6682926	ERS13545378	Cutaneous lesion	1	1	0	7	1	31		
6670223	ERS13545379	Cutaneous lesion	22	22	22	665.05	0.96	20	18.44	−1.56
6687073	ERS13545380	Cutaneous lesion	23	22	12	34.35	0.97	24	23.40	−0.60
6664375	ERS13545381	Cutaneous lesion	22	22	22	392.59	0.96	21	19.30	−1.70
6666575	ERS13545382	Cutaneous lesion	22	22	22	173.09	0.96	21	20.25	−0.75
6642566	ERS13545383	Cutaneous lesion	22	22	22	294.91	0.96	15	19.72	4.72
6642564	ERS13545384	Cutaneous lesion	22	22	22	90.91	0.96	21	20.61	−0.39
6662714	ERS13545385	Cutaneous lesion	22	22	22	277.64	0.96	18	19.80	1.80
6653234	ERS13545386	Plasma	0	0	0	0	0	37		
6669393	ERS13545387	Cutaneous lesion	22	22	22	456.73	0.96	21	19.02	−1.98
6665712	ERS13545388	Cutaneous lesion	0	0	0	0	0	26	25.90	−0.10
6747356	ERS13545389	Cutaneous lesion	0	0	0	0	0	NEG		
6747790	ERS13545390	Rectal/pharyngeal	0	0	0	0	0	NEG		
6651064	ERS13545391	Cutaneous lesion	22	22	22	116	0.97	22	20.18	−1.82
6650976	ERS13545392	Rectal exudates	22	22	22	170.27	0.96	14	19.94	5.94
6669392	ERS13545393	Cutaneous lesion	0	0	0	0	0	NEG		
6642550	ERS13545394	Cutaneous lesion	23	22	22	264.26	0.95	16	19.54	3.54
6656789	ERS13545395	Rectal exudates	0	0	0	0	0	NEG		
6669307	ERS13545396	Cutaneous lesion	22	22	20	53.59	0.97	23	20.90	−2.10
6655994	ERS13545397	Cutaneous lesion	22	22	22	112.5	0.96	23	20.20	−2.80
6651585	ERS13545398	Pharyngeal exudates	22	22	22	85.23	0.97	21	20.31	−0.69
6669306	ERS13545399	Cutaneous lesion	22	22	22	139.5	0.96	21	20.08	−0.92
6655713	ERS13545400	Cutaneous lesion	22	22	22	104.36	0.96	19	20.23	1.23

a The table shows the code sample number with its corresponding ENA access, the area from which the sample is taken, the total SNPs that have been called, SNPs markers of the outbreak, and SNPs markers >30X depth, mean frequency, and coverage statistics, as well as Ct results, both experimental and statistically inferred with difference. Negative samples (NEG) or above Ct 30 were excluded from this Ct prediction. The 10 samples included in each run are split by shading.

The total turnaround time required by our multiple-amplicon nanopore sequencing method (multiplex PCR, library preparation, sequencing run, and flow cell wash to be reused) was 5 h. Cost per specimen was adjusted to 14 euros due to the decision to reuse one single flow cell up to 12 times. Costs for standard diagnostic MPXV RT-PCR were 18 euros/specimen. We must acknowledge that our analysis times did not accelerate the response time offered by the RT-PCR-based scheme applied to MPXV diagnosis in our institution. However, we must bear in mind that a positive result from the commercial PCRs just indicates the presence of MPXV, without being able to differentiate whether it corresponds to the outbreak strain or any other strain. It is true that in the current epidemiological scenario, with most cases out of Africa corresponding to the same clade B1, the generic PCR result may be sufficient to infer that we are detecting the outbreak strain. However, some sequences different from clade B1, belonging to clade A1, have also been recently identified in Europe and the United States (PHE technical briefing 5), corresponding to other independent exportation events. We should therefore be prepared to more precisely discriminate the strains from forthcoming cases. Moreover, our study intended to select the current MPXV outbreak just as a proof of concept to evaluate a strategy that may offer an optimized fast track of relevant public health strains in a wider spectrum of epidemiological alarms involving other viruses/bacteria.

Nanopore sequencing of multiple amplicons harboring signature SNPs escapes the targeting limitations of strain-specific PCRs and offers a powerful alternative to systematic whole-genome analysis, paving the way to real-time genomic epidemiology and making immediate intervention possible to finally optimize transmission control.

### Data availability.

The data supporting the findings of this study (FastQ files) were deposited at ENA (https://www.ebi.ac.uk) under the project accession number PRJEB56588; the accession numbers of the sequenced strains used in the study can be found in Table 2.
